# The combined consumption of fresh/minimally processed food and ultra-processed food on food insecurity: COVID Inconfidentes, a population-based survey

**DOI:** 10.1017/S136898002300054X

**Published:** 2023-07

**Authors:** Hillary Nascimento Coletro, Luiz Antônio Alves de Menezes-Júnior, Raquel de Deus Mendonça, Adriana Lúcia Meireles, George Luiz Lins Machado-Coelho, Mariana Carvalho de Menezes

**Affiliations:** 1 Universidade Federal de Ouro Preto, Research and Study Group on Nutrition and Public Health (GPENSC), School of Nutrition, Ouro Preto, Minas Gerais, Brazil; 2 Universidade Federal de Ouro Preto, Department of Clinical and Social Nutrition, Research and Study Group on Nutrition and Public Health (GPENSC), School of Nutrition, Ouro Preto, Minas Gerais, Brazil; 3 Universidade Federal de Ouro Preto, Epidemiology Laboratory, School of Medicine, Ouro Preto, Minas Gerais, Brazil

**Keywords:** Food insecurity, Ultra-processed foods, NOVA classification, COVID-19 pandemic

## Abstract

**Objective:**

To investigate whether the combined consumption of fresh/minimally processed and ultra-processed food is associated with food insecurity (FI) during the COVID-19 pandemic.

**Design:**

Cross-sectional observational study was derived from a survey using a population-based search of a complex sample. FI was assessed using the validated Brazilian Food Insecurity Scale. Food consumption was assessed using a qualitative FFQ and the NOVA classification for fresh/minimally processed food and ultra-processed food. A scoring system was used to evaluate combined food consumption according to the extent and purpose of processing, considering the weekly consumption of the two groups (according to the NOVA classification). Higher punctuation reflects worse diet quality (higher consumption of ultra-processed foods and lower consumption of fresh/minimally processed foods). A theoretical causality model was constructed using a directed acyclic graph, and multivariate analysis was performed using Poisson regression to test the association between FI and food consumption.

**Setting:**

Ouro Preto and Mariana, Brazil, between October and December 2020.

**Participants:**

An epidemiological household survey was conducted with 1753 individuals selected through a stratified and clustered sampling design in three stages.

**Results:**

Those with food consumption scores in the fourth quartile had a 60 % higher prevalence ratio (PR) for FI (PR: 1·60 and 95 % CI: 1·06 - 2·40). Also, the increased consumption of fresh/minimally processed foods and low consumption of ultra-processed foods presented a 45 % lower prevalence ratio of FI (PR: 0·55 and 95 % CI: 0·40 – 0·80).

**Conclusion:**

These results indicate an inverse association between FI and diet quality.

Although the ‘Sustainable Development Goals’ indicate the priority to eradicate poverty and hunger by the year 2030^(1)^, the number of people affected by hunger is increasing. In 2020, between 720 and 811 million people worldwide faced hunger, and 30·4 % suffered from moderate or severe food and nutrition insecurity^(2)^. In Brazil, malnutrition impacted up to 5·2 million people between 2015 and 2017^(3)^. In 2020, during the COVID-19 pandemic, the number of Brazilians facing hunger increased to 19 million, while 116·8 million Brazilians suffered from food insecurity (FI)^(4)^.

The eradication of hunger and malnutrition can be achieved through food and nutrition security, which is defined as having the right to consistent and permanent access to sufficient, quality food without compromising access to other essential needs and based on health-promoting food practices respecting cultural diversity, which are environmentally, culturally, economically and socially sustainable^(5)^.

An important aspect of FI is ensuring access to food with nutritional quality^(5)^. However, we have to consider that the nutritional transition has been followed by a reversal of traditional eating habits, which used to be characterised by the high consumption of natural and home-cooked foods, and now are being replaced by the consumption of ready-made foods with large industrial processes, collectively referred to as ultra-processed foods (UPF) with exaggerated addition of salt, sugar, fat and substances exclusively used by industry and poor micronutrients (i.e. vitamins and minerals)^(6)^. A significant increase in the consumption of UPF is expected due to their lower prices^(7)^, convenience^(8)^, palatability^(9)^, storage^(6)^ and easy access during this health crisis^(10)^. Increased consumption leads to a deficient intake of vitamins, minerals and protein, and a high intake of saturated fat, sugar, salt, strongly flavoured ingredients and chemical additives, leading to an increase in nutritional insecurity^(11,12)^, negative health effects^(13–16)^ and contributing to an unsustainable food system^(17)^.

Food has recently become an important public health topic; as such, it is necessary to understand that nutrition cannot be defined solely as energy and nutrient intake^(6)^; this approach minimises ‘nutrition’ by overlooking the wider socio-cultural aspects of meals. It is important to consider all dimensions of food security, including utilisation, which refers to the intake of safe food in sufficient quantities and also covers nutritional knowledge and food choices^(18)^. Furthermore, it is fundamental to consider the quality of the food through the degree of industrial processing. Thus, the objective of this study was to investigate whether the combined consumption of fresh/minimally processed food and UPF was associated with FI during the COVID-19 pandemic in Brazil.

## Material and methods

### Study design and location

The present study was carried out in two cities in Brazil via an epidemiological household survey led in three stages between October 2020 and December 2020. These data were derived from the ‘Epidemiological surveillance of COVID-19 in the Inconfidentes Region/MG’, previously described by Meireles *et al.* ^(19)^.

The research was conducted in the cities of Ouro Preto and Mariana, which, according to the 2010^(20)^ demographic census, have a total of 108 170 people living in the urban area, distributed in 17 753 households in Ouro Preto and 14 078 in Mariana. Ouro Preto has a municipal human development index of 0·741, and Mariana has an municipal human development index of 0·742^(20)^.

### Study population and sampling

Residents over the age of 18 and living in the urban areas of Ouro Preto and Mariana were considered eligible for this study.

The required sample size was calculated using the OpenEpi tool, with the population estimated by the demographic census^(20)^ for urban areas, 95 % confidence level, design effect equal to 1·5, SARS-CoV-2 infection (estimate of 3 % to 10 %) and precision. Additionally, a 20 % recomposition was considered to account for any loss.

The sample design was performed by conglomerates in three stages: census sector, household and resident. This design was based on large national household surveys, such as the National Household Sample Survey^(21)^; Family Budget Survey^(22)^; ‘Saúde em Beagá’ survey^(23)^, and more recently, the ‘EPICOVID19’^(24)^. Therefore, in the study design, the census sectors were considered primary sampling units, selected with probability proportional to the number of households, using the number of households obtained from the 2010 population census as a measure^(20)^. Sample stratification was performed before choosing the primary units, considering the average income according to data from the 2010 demographic census^(20)^ to ensure diverse income strata (< 1 minimum wage (MW), 1 to 3 MW and ≥ 4 MW). The secondary sampling units were households systematically selected using the updated listing of existing household units in the primary sampling units (selected census sectors). After the selection of the census sectors, the household selection interval (k) was calculated for systematic sampling according to the following formula: k = Ni/(xi/ni), where Ni is the total number of households in the census sector, xi is the sample size and ni is the number of households selected in the census sector. Thus, a proportional number of homes per sector was obtained, covering the entire geographical area. The third sampling unit consisted of individuals selected from simple random sampling. A list of all adult residents was made from each household selected, and a simple random drawing of one resident participating in the research was carried out.

Based on the sample calculation, 1464 individuals should have been interviewed in the two cities evaluated^(19)^. During the data collection process, we approached 5252 households, of which 1912 (36·4 %) were closed, 267 (5·1 %) were randomly selected, but the resident was absent and 1079 (20·5 %) refused to participate. Therefore, 1789 (34·0 %) households agreed to participate in the study. Of these, twenty-five did not complete the interview, and eleven did not respond to the Brazilian Food Insecurity Scale (EBIA). Ultimately, 1753 individuals were included in this study, representing adult residents in the urban areas of two Brazilian cities.

### Data collection

Data collection occurred from October to December 2020. The process began by approaching households. If the individuals accepted to participate in the research, the names of all household residents aged 18 years or older with the cognitive function to participate in the interview were listed in a digital drawing application that randomly chose one resident for the face-to-face interview. Data collection was conducted on Friday, Saturday and Sunday to facilitate resident participation. In the previous week, the research team recruited households in pre-selected census sectors.

The interviews were conducted by a trained team whose health was tracked through periodic evaluations, including testing for anti-SARS-CoV-2 antibodies before beginning each stage of the survey. In addition, all recommended national protocols against Coronavirus were adopted.

The face-to-face interviews lasted approximately 40 min, using the DataGoal® application. The questionnaire gathered socio-demographic and economic data, food consumption and EBIA.

### Outcome variable: food insecurity

A validated tool was used to assess FI. The EBIA is a psychometric scale that directly measures household experience with FI over the past 3 months. The subjective assessment included the following: perception of food access, food availability at home, concern about the possibility of lack of food, impairment of food quality and impairment of the quantity of food for adults and children (under 18 years of age)^(5)^.

The EBIA is an instrument composed of eight questions targeted at adults and six more when a minor resident lives in the household. Based on the sum of the answers, it is created a scoring system that ranged from 0 to 14 points so that, considering only adult residents, a household with a score of zero indicates food security, scores of 1–3 points indicate mild FI, 4 –5 points indicate moderate FI and 6 to 8 points indicate a situation of severe FI present in the household. However, when there was a resident under 18 years of age in the household, a score of 1–5 points indicated mild FI, 6–9 points indicated moderate FI and 10–14 points indicated severe FI^(5)^. In addition to using the categories mentioned above, the present study also reported the data in its dichotomised form, that is, the presence of food security *v*. FI, across all levels (mild, moderate and severe) in the household.

### Exposure variable: food consumption

Food consumption was assessed using a qualitative FFQ, a tool that presents a list of foods and/or preparations and frequency categories, referring to the consumption of eighteen foods, widely consumed by Brazilians^(22)^, over the last 3 months. The frequency of food consumption was reported on weekdays, with five possible answers: (i) never; (ii) 1–2 d/week; (iii) 3–4 d/week; (iv) 5–6 d/week and (v) every day (including Saturday and Sunday).

FFQ foods were analysed according to the degree of processing: (i) fresh/minimally processed foods and (ii) UPF, classified according to the Dietary Guidelines for the Brazilian Population^(6)^ and NOVA classification^(25)^.

NOVA methodology is an important metric that classifies foods based on industrial processing and its implications for dietary patterns and human health^(25)^. NOVA classifies foods into four groups: (i) fresh or minimally processed foods; (ii) culinary ingredients; (iii) processed foods and (iv) UPF^(6)^.

We used two NOVA categories: fresh/minimally processed and UPF, to indicate healthy and unhealthy eating patterns. In both groups, foods that are commonly consumed by Brazilians were investigated^(26)^. The group of fresh/minimally processed foods represents foods with minimal processing techniques to make them suitable for storage^(6)^, and this group composed of beans, nuts, vegetables, dark green vegetables, fruits, red meat, chicken, fish and eggs; we considered the consumption of soft drinks, chocolate drinks, artificial yogurt, cookies, packed snacks, instant noodle, frozen products, processed meat, sweetbreads and other sweets as UPF (i.e. foods with the highest degree of industrial processing, formulated with several techniques and many ingredients, including substances for industrial use^(6)^).

To test the hypothesis that high weekly consumption of UPF is associated with FI during the COVID-19 pandemic, a scoring system was used considering the weekly consumption of fresh/minimally processed foods and UPF, as proposed by Francisco *et al.* ^(27)^. The score ranged from 0 to 4 points, depending on food and weekly consumption frequency. The score was calculated inversely so that higher punctuation indicates a worse diet quality; for the daily consumption of fresh/minimally processed foods and rare or never consumption of UPF, punctuation was the minimum score (zero). The highest score (four points) was received by the rare or never consumption of fresh/minimally processed foods and daily consumption of UPF (Table 1, Supplementary Material). The total score ranges from 0 (indicating the best diet quality) to 53 points (indicating the worst diet quality). The total score was categorised into quartiles of distribution.

**Table 1:** Socio-demographic characteristics of the sample, COVID Inconfidentes 2020

Sample description	Total	95 % CI
Sex†		
Male	48·0	40·9, 55·2
Female	52·0	44·8, 59·1
Age†		
18–34 years	35·6	31·2, 40·4
35–59 years	45·6	41·1, 50·2
≥ 60 years	18·8	15·4, 22·5
Marital status†		
Married	46·9	40·9, 52·9
Not married*	53·1	47·1, 59·1
Number of people sharing the same household‡	3·7	3·6, 3·9
Skin colour†		
White	25·7	20·9, 31·3
Brown	48·0	41·6, 54·5
Black	20·7	16·0, 26·5
Indigenous, yellow and not reported¶	5·6	4·0, 7·7
Education†		
Never attended school	1·5	0·7, 3·4
1–9 years	29·5	25·1, 34·2
> 9 years	69·0	64·1, 73·4
Family income†,§		
≤ 2 MW	41·1	35·6, 46·8
> 2–≤ 4 MW	31·9	26·8, 37·4
> 4 MW	27·0	22·0, 32·6
Workins status†		
Working	52·1	47·3, 56·8
Not Working||	47·9	43·2, 52·7
Change in income after the COVID – 19 pandemic†		
Yes. It has reduced	36·9	31·4, 42·7
Yes. It has increased	7·6	4·5, 12·6
No change	55·5	51·0, 59·9

* Not married: widowed, divorced or single.

† Values expressed as proportion and 95 % CI.

‡ Values expressed as mean and 95 % CI.

§ MW: minimum wage of the year when data collection occurred, 2020 – BRL 1045.00 or about USD 194.

|| Not working: individuals who were not working during the data collection period and with no employment income.

¶ Not reported: In the case of the participant not being able to report the colour of his skin, his answer was allocated as not reported (1·5 % of the answers).

In addition, to test the second hypothesis that consumption of fresh/minimally processed foods can have a protective effect on FI, considering the consumption of UPF at the same time, four food consumption patterns were created based on the extent and purpose of food processing and the weekly consumption frequency of all the foods included in these groups. First, two explanatory variables were created: fresh/minimally processed foods and UPF, from the sum of the weekly consumption frequency of each food group (e.g. for fresh/minimally processed foods = weekly frequency of fruit consumption + weekly frequency of egg consumption + weekly frequency of vegetable consumption+…). Subsequently, these two variables were categorised as below the average weekly consumption frequency, referring to lower weekly consumption and above or equal to the average weekly consumption frequency, referring to higher weekly consumption. Thus, a combined assessment of different food consumption patterns was performed: (i) low consumption of fresh or minimally processed foods and low consumption of UPF; (ii) low consumption of fresh/minimally processed foods and high consumption of UPF; (iii) high consumption of fresh/minimally processed foods and high consumption of UPF and (iv) high consumption of fresh/minimally processed foods and low consumption of UPF. Then, the following hypotheses were assessed: whether the high consumption of UPF and the low consumption of fresh/minimally processed food have a greater effect on FI and whether the high consumption of fresh or minimally processed food and the low consumption of UPF have a protective effect on FI.

### Covariates

Socio-demographic variables were investigated to describe the sample and explore possible confounding factors in the association analysis between food consumption and FI.

The socio-demographic variables investigated were sex, age (age:18–34 years old, 35–59 years old, and 60 years old or more), marital status (having a partner or not), number of people sharing the same household, skin colour (white, black, brown, indigenous, yellow and not reported), education (never attended to school, 1–9 years of study or more than 9 years of study), family income (up to two MW, 2–4 MW or more than four MW), working status (working or not working), change in income after the COVID-19 pandemic (reduced, increased or did not change) and perceived change in food prices, in general (I did not notice any change, yes, the prices increased and yes, food prices have decreased).

### Statistical analysis

First, the sample weight of each selected unit (census sector, household, and individual) was calculated separately for each city to adjust the natural weight of the design and correct problems caused by the absence of or refusal to respond. The calibration of the natural expansion factors consisted of estimating new weights for each participant in the sample. The probability of selecting the census sector in each city in the sample is given by


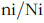




where

ni = number of census sectors in the sample selected from the city

Ni = total number of census sectors in the entire city

The probability of the household in census sector ‘j’ being selected was obtained from the following expression:


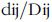




where

‘dij’ is the number of sampled households.

‘Dij’ is the number of households in the sector.

The probability of each individual residing in the selected household was calculated by 1/ (number of residents aged 18 years or older in the household)^(28)^.

The analyses were performed using Stata software version 15.1 (Stata Corporation), using the command ‘svy’, which considers a complex sample design. For the socio-demographic variables and the prevalence of FI, we used a proportion and a 95 % CI to describe the data.

To assess the relationship between food consumption and different levels of FI, the average score and its respective 95 % CI were reported for individuals with mild, moderate and severe FI.

Multivariate analysis was performed using Poisson regression with the prevalence ratio (PR) and respective 95 % CI for binary outcomes to verify the association between FI and food consumption. To verify the combined consumption of fresh/minimally processed and UPF, we used as reference the pattern indicating low consumption of fresh/minimally processed foods and low consumption of UPF.

**Fig. 1:**
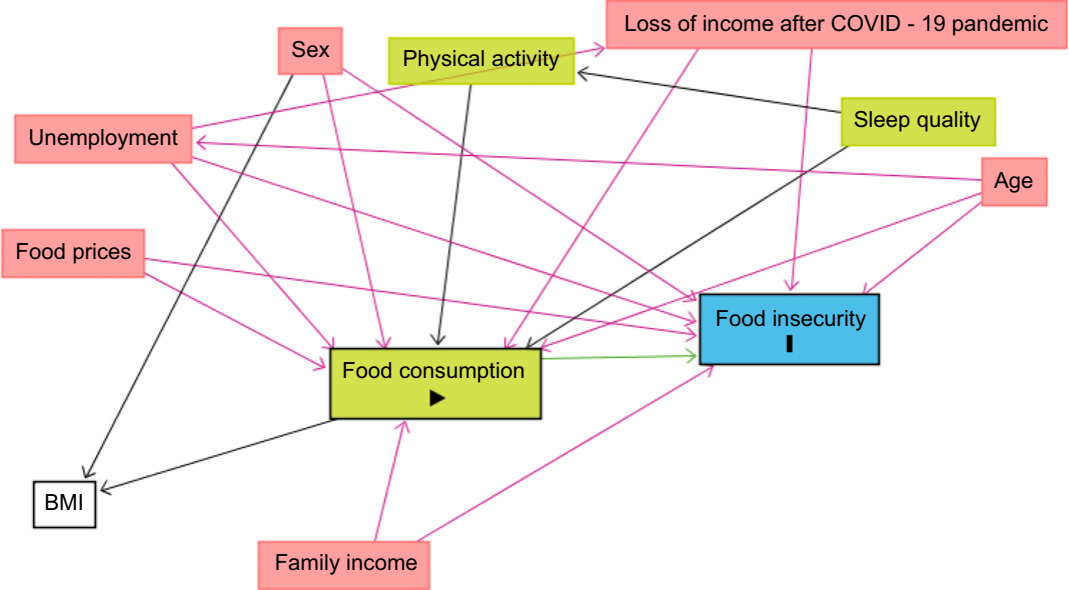
Directed Acyclic Graph (DAG) for the food consumption score and food insecurity, with possible confounding variables, COVID Inconfidentes 2020

To select adequate adjustment variables, a theoretical causality model was constructed through a directed acyclic graph considering the exposure (food consumption score), outcome (FI) and possible confounding variables. The online software Dagitty version 3·2 was used (Textor and Hardt, 2011). The causal connections are represented by arrows (Fig. 1). Each variable in the directed acyclic graph was chosen based on the recent literature and scientific evidence^(29–32)^. A minimum set of adjustment variables was defined to avoid unnecessary adjustments, spurious associations and estimation errors. Furthermore, the parameters for model evaluation were appreciated (Prob F < 0·001 and goodness-of-fit test), indicating that the model’s variables were appropriate for the analysis. Considering the directed acyclic graph results, the multivariate model was adjusted for sex, age, family income, loss of income after the COVID-19 pandemic, employment status and change in food prices.

## Results

**Fig. 2:**
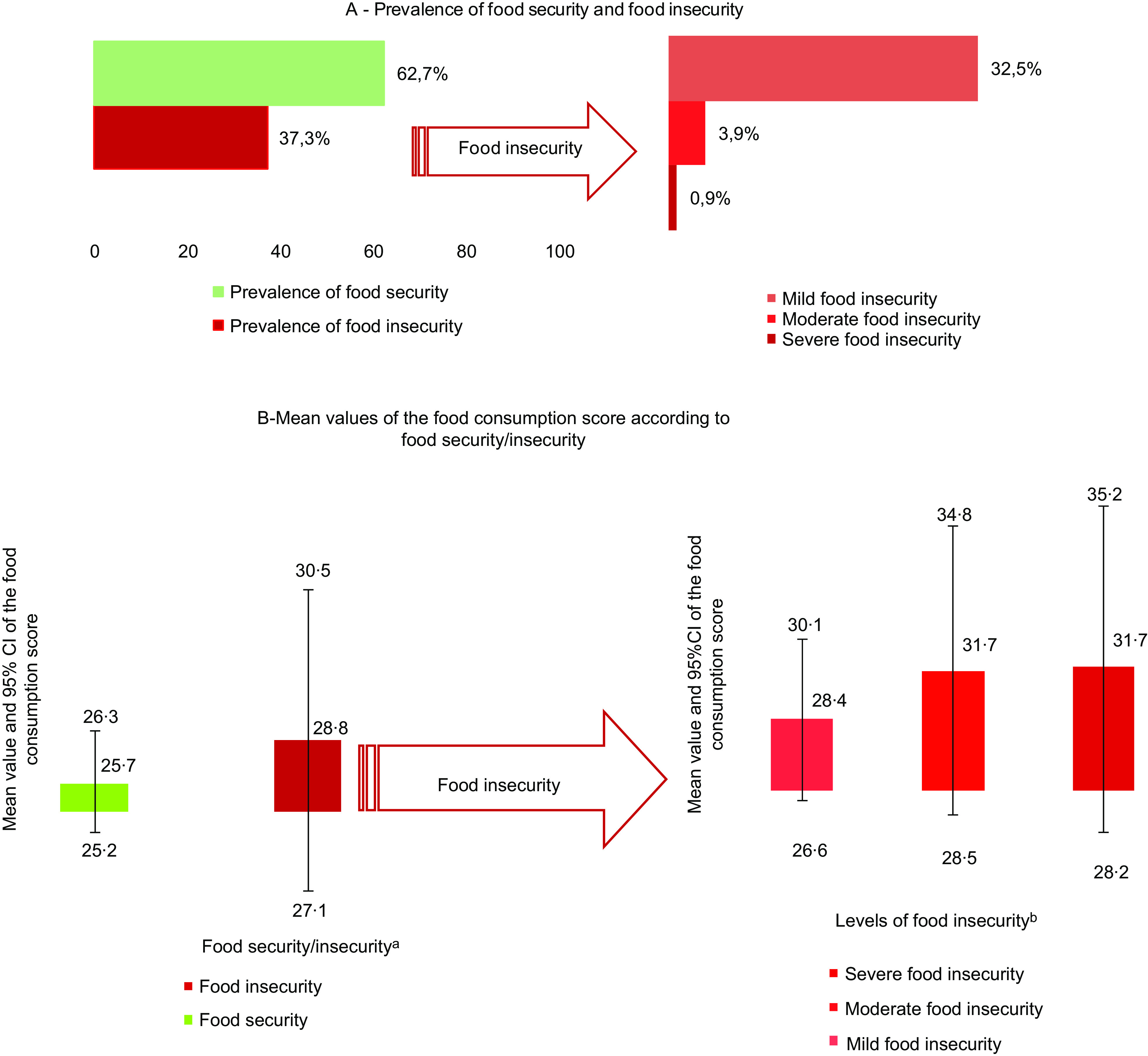
Prevalence of food security/insecurity and description according to the degree of food consumption processing, COVID Inconfidentes 2020

Among the participants, 37·2 % were in a situation of FI; among them, 32·5 % were considered to be in the mild FI category (Fig. 2).

Socio-demographic characteristics are shown in Table 1. Among the participants, the majority were female, between the ages of 35 and 59, unmarried, brown, with more than nine years of education and family income below two MW. In addition, most reported that they were working during the interview period, with no change in income.

The mean food consumption score was 26·9 points (95 % CI: 26·0, 27·7) among the participants. Figure 2 describes the mean food consumption score according to food security (mean: 25·7; 95 % CI: 25·2, 26·3)/insecurity (mean: 28·8; 95 % CI: 27·1, 30·5). Individuals with FI had higher scores on the food consumption scale, indicating worse diet quality than adults in food security situations. The score was especially higher for adults suffering from moderate FI (mean: 31·7; 95 % CI: 28·5, 34·8) and severe FI (mean: 31·7; 95 % CI: 28·2, 35·2) compared to mild insecurity (Fig. 2).

Multivariate regression analysis of the relationship between food consumption according to the degree of processing and the presence of FI (Table 2) revealed that those with food consumption scores in the fourth quartile (indicating higher consumption of UPF and lower consumption of fresh and minimally processed foods) had a 60 % greater PR for FI (PR:1·60 and 95 % CI: 1·06, 2·40).

**Table 2:** Prevalence ratio (PR) and 95 % CI for the association between food consumption score and food insecurity, COVID Inconfidentes 2020

Quartile of the food consumption score	Min. and max. score values	Prevalence		Unadjusted analysis		Adjusted analysis*		
		%	95 % CI	RP	95 % CI	RP	95 % CI	*P* trend§
								0·008
Q1†	6–21	26·1	21·2, 31·6	ref	–	ref	–	
Q2	22–26	21·3	17·6, 25·6	1·14	0·62, 1·87	1·06	0·69, 1·64	
Q3	27–31	26·2	21·9, 30·9	1·55	0·98, 2·46	1·47	0·93, 2·23	
Q4‡	32–53	26·4	21·8, 31·7	1·75	1·21, 2·54	1·60	1·06, 2·40	

* Adjusted analysis by the following minimum set of variables: sex, age, family income, loss of income after the COVID-19 pandemic, working status and change in food prices.

† Quartiles of food consumption score distribution: Q1 are the lowest values of the score, characterised by the highest consumption of fresh and minimally processed foods and lowest consumption of ultra-processed foods.

‡ Q4 are the highest values of the score, characterised by the lowest consumption of fresh and minimally processed foods and the highest consumption of ultra-processed foods.

§ Linear trend tests; The food and nutrition insecurity was used as the outcome.

**Fig. 3:**
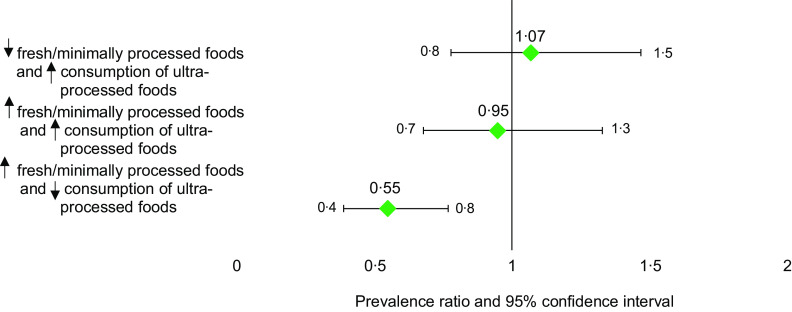
Prevalence ratio (PR) and 95 % CI for the association between the combined consumption of fresh/minimally processed and ultra-processed food and food insecurity, COVID Inconfidentes 2020

Furthermore, through multivariate regression analysis with an interaction between the combined consumption of fresh/minimally processed and UPF consumption and FI (Fig. 3), it was possible to identify four eating patterns in which the high consumption of fresh/minimally processed foods and low consumption of UPF were associated with a lower PR of FI, indicating that individuals with this eating pattern had a 45 % lower PR for FI.

## Discussion

To our knowledge, this is the first study to report that low consumption of fresh/minimally processed foods and high consumption of UPF was associated with a higher prevalence of FI; in the same way, the combined occurrence of two positive eating habits (higher frequency of fresh/minimally processed foods and lower frequency of UPF consumption) was associated with a lower likelihood of experiencing FI during the COVID-19 pandemic, contributing to the growing literature on the harmful effects of the highest degree of industrial food processing.

The consumption of UPF represents an important and growing part of the world food supply and accounts for approximately half of the total calories consumed in the USA^(33)^, UK^(15)^ and Canada^(34)^. For example, in Brazil, it was observed that between 2008 and 2018, there was a substantial decrease in the consumption of grains, cereals and fruits, and an increase in the consumption of sandwiches and pizzas^(26)^, with UPF providing an average of 20·4 % of the daily calories^(35)^.

Food choices are not only determined by physiological and nutritional needs but also by the influence of environmental factors such as accessibility, availability, affordability and cultural factors^(36)^. During the pandemic, social distancing measures and governmental blockades have imposed new routines and lifestyles worldwide, mainly affecting the food production chain and eating habits^(37)^. The production of fresh and minimally processed food, mostly carried out by family farmers, was affected in such a way that its production chain was limited not only by food distribution and logistical difficulties but also by trade, with a drastic reduction in the sale of less commercialised food, giving way to a greater purchase of processed food and UPF^(38)^. Food processing has become a central force shaping the hegemonic agroindustrial food system^(39)^.

There is evidence for an increase in the exposure of different population groups to an environment that favours the consumption of UPF, not only during the COVID-19 crisis but also due to its high availability, convenience, palatability^(9)^, low cost and aggressive marketing^(13)^. However, the pandemic worsened the economic crisis in several countries with an increase in unemployment rates and changes in family income, contributing to changes in consumption patterns^(40)^. As a result, a worsening of the nutritional profile of diets is expected, followed by an increase in FI, especially in the dimension of availability and access to food.

The literature shows a strong relationship between FI and indicators of unhealthy eating^(41)^. The literature on the consumption of UPF and FI, specifically is still incipient. A study in Canada with 15 909 children and adults found that the percentage of energy from the UPF was strongly related to the severity of FI^(12)^. In addition, a study conducted in the Philippines found similar results, demonstrating that UPF in soft drinks, flavoured juice drinks and energy drinks were associated with moderate and severe levels of FI^(42)^.

A systematic review found that FI among adults was associated with low consumption of vegetables, fruits and dairy products and a lower intake of vitamins A and B6, Ca, Mg and Zn^(43)^. It is important to highlight that the regular consumption of fresh foods can be considered a protective factor against FI in its nutritional dimension, as demonstrated in this study in agreement with Araújo *et al.* ^(44)^, because fresh foods provide several nutrients^(45)^ and can serve a prophylactic function.

However, evidence suggests that food prices have varied to favour the consumption of processed and UPF in Brazil and several other developing countries^(46)^. Previous studies have reported that households with FI face difficulty accessing affordable healthy foods^(30,32)^. In addition, national surveys have shown that families in situations of greater vulnerability have more access to sugary drinks, cookies, packaged and frozen foods and other UPF instead of fresh foods such as fruits, vegetables, whole grains and lean meats^(47)^. In this regard, these individuals tend to have a higher prevalence of FI and worse health outcomes.

Therefore, it is well-known that a diet rich in fresh foods and nutrients is essential for promoting health and commensality, while a diet rich in UPF, deficient in vitamins, minerals and proteins, is capable of worsening quality of life, food and nutritional security, as well as being related to all causes of mortality^(14)^. Thus, there is a strong need to reformulate public health policies and include measures to ensure food security, not only in quantity but also in quality, considering food processing (based on the Food Guide for the Brazilian Population^(6)^).

This study highlights the novelty of food consumption from the NOVA classification and considers FI during the COVID-19 pandemic; however, previous studies support our findings. Leung *et al.* ^(11)^ found that the consumption of highly palatable foods, such as snacks and sugar-sweetened beverages, was higher, while fruit and vegetable intake was lower among adults with FI; thus, food-insecure adults in this sample consumed 12 % fewer servings of vegetables than food-secure individuals. Between 2010 and 2018, data from the Eating and Activity over Time study^(48)^ collected from 1568 individuals noted that FI was associated with poorer diet quality, characterised by lower consumption of vegetables and whole grains and more sugar-sweetened beverages. Therefore, knowledge about food consumption in food-insecure families is highlighted so that it is possible to map and create strategies to promote health and adequate food supply in a way that involves food choices, whether economic or spatial^(38)^.

However, this study has some minor limitations. First, this is a cross-sectional study, which does not allow us to establish causal inferences. Despite the study being conducted in a period when FI increased in Brazil^(4)^ and the availability of UPF also increased^(13)^, our study does not allow us to assess changes over time and the bidirectional relationship of the data, since food consumption can explain FI and also can be the outcome caused by FI. In this sense, there is a possibility that the prevalence described in this study was the result of events prior to the COVID-19 pandemic. The literature shows that even before the COVID-19 pandemic, food-insecure households had less availability of fresh/minimally processed foods, such as fruits, vegetables, legumes and beans, as demonstrated by Araújo *et al.*, assessing 2817 individuals in Belo Horizonte, Brazil^(44)^. In Brazil, a study in the Amazon that sought to assess food consumption and FI noted that fresh/minimally processed foods, such as fruits and vegetables, were considered unaffordable^(49)^. Using data from the 2007–2016 National Health and Nutrition Examination Survey, Leung *et al.* ^(50)^ found that severe FI was associated with higher consumption of UPF in the USA. Therefore, it is likely that this scenario was already present prior to the COVID-19 pandemic and may have worsened. Second, food consumption was estimated using a non-validated questionnaire, and in that case, the study may be subject to measurement error; however, the FFQ contemplated the foods most consumed by the Brazilian population according to the National Family Budget Survey^(22)^. Third, the outcome studied here, FI, is composed of several dimensions that are not assessed by a single method. However, the scale chosen is used internationally and has been validated in Brazil. It is important to consider that to be eligible for the interview and the EBIA, the resident selected had to be 18 years old or older, not necessarily the head of the household.

We can also highlight some potentials of the present work. The topic of food consumption, considering industrial processing and how this affects the food system, food consumption and FI, is recent. In addition, the assessment by household surveys during the COVID-19 pandemic provides great robustness to the study. Face-to-face interviews allowed greater accuracy of the information and promoted methodological strength, while probabilistic sample selection and sample weight provided statistical power to the study, as well as internal and external validity. During the pandemic, most studies that assessed FI were conducted online or in convenient samples, which can lead to biased results because the population at risk for FI is usually people with lower income and thus may not respond to online interviews, especially in low- and middle-income countries such as Brazil.

In conclusion, the present study revealed an inverse association between FI and diet quality based on the NOVA classification. Therefore, we encouraged a diet based on the consumption of food with a lower degree of processing, as recommended by the Dietary Guidelines for the Brazilian population. Future work requires epidemiologic studies that investigate this relationship on a longitudinal basis and studies that explore the degree of processing and the development of FI in all its dimensions.

## Supplementary material

For supplementary material accompanying this paper visit https://doi.org/10.1017/S136898002300054X. click here to view supplementary material
