# Supplementary material for: The combined consumption of fresh/minimally processed food and ultra-processed food on food insecurity: COVID Inconfidentes, a population-based survey
Source: Public Health Nutr. 2023 Mar 15;26(7):1414–23. doi: 10.1017/S136898002300054X (PMC10346086; doi:10.1017/S136898002300054X)
Supplement: Supplementary file 1 [file S136898002300054Xsup001.docx]

| **Points** | **0** | **1** | **2** | **3** | **4** |
| --- | --- | --- | --- | --- | --- |
| **Fresh/minimally processed** | | | | | |
| **Beans^a^** | Daily | 5 to 6 days per week | 3 to 4 days per week | 1 to 2 days per week | Never or almost never |
| **Nuts^b^** | Daily | 5 to 6 days per week | 3 to 4 days per week | 1 to 2 days per week | Never or almost never |
| **Vegetables^c^** | Daily | 5 to 6 days per week | 3 to 4 days per week | 1 to 2 days per week | Never or almost never |
| **Dark green vegetables^d^** | Daily | 5 to 6 days per week | 3 to 4 days per week | 1 to 2 days per week | Never or almost never |
| **Fruit** | Daily | 5 to 6 days per week | 3 to 4 days per week | 1 to 2 days per week | Never or almost never |
| **Red meat** | Daily | 5 to 6 days per week | 3 to 4 days per week | 1 to 2 days per week | Never or almost never |
| **Chicken** | Daily | 5 to 6 days per week | 3 to 4 days per week | 1 to 2 days per week | Never or almost never |
| **Fish** | Daily | 5 to 6 days per week | 3 to 4 days per week | 1 to 2 days per week | Never or almost never |
| **Egg** | Daily | 5 to 6 days per week | 3 to 4 days per week | 1 to 2 days per week | Never or almost never |
| **Ultra-processed** | | | | | |
| **Soft drinks** | Never or almost never | 1 to 2 days per week | 3 to 4 days per week | 5 to 6 days per week | Daily |
| **Chocolate drink and artificial yogurt** | Never or almost never | 1 to 2 days per week | 3 to 4 days per week | 5 to 6 days per week | Daily |
| **Cookies** | Never or almost never | 1 to 2 days per week | 3 to 4 days per week | 5 to 6 days per week | Daily |
| **Packed snack** | Never or almost never | 1 to 2 days per week | 3 to 4 days per week | 5 to 6 days per week | Daily |
| **Instant noodle** | Never or almost never | 1 to 2 days per week | 3 to 4 days per week | 5 to 6 days per week | Daily |
| **Frozen products^e^** | Never or almost never | 1 to 2 days per week | 3 to 4 days per week | 5 to 6 days per week | Daily |
| **Processed meat^f^** | Never or almost never | 1 to 2 days per week | 3 to 4 days per week | 5 to 6 days per week | Daily |
| **Sweet breads^g^** | Never or almost never | 1 to 2 days per week | 3 to 4 days per week | 5 to 6 days per week | Daily |
| **Sweets^h^** | Never or almost never | 1 to 2 days per week | 3 to 4 days per week | 5 to 6 days per week | Daily |

Note: ^a^Beans or soy, peas, lentils, chickpeas; ^b^Nuts or chestnuts, peanuts, walnuts, almonds, macadamia, among others; ^c^lettuce, tomato, carrot, chayote, eggplant, zucchini; ^d^spinach, kale, watercress, arugula; ^e^pizza, nuggets, french fries; ^f^hamburger, sausage, bologna, salami, ham, turkey breast; ^g^Loaf bread, hamburger or hot dog bread or other sweet bread; ^h^candy, gum, ice cream, gelatin and chocolate

**Table 1 -** Score to analyze the consumption of fresh/minimally processed and ultra-processed foods, COVID INCONFIDENTES 2021.
